# From MINI to Meaningful Change—A German Pilot Study to Improve Patient Outcomes in End-of-Life Care

**DOI:** 10.3390/healthcare13162024

**Published:** 2025-08-16

**Authors:** Jana Sophie Grimm, Alina Kasdorf, Raymond Voltz, Julia Strupp

**Affiliations:** 1 Department of Palliative Medicine, Faculty of Medicine and University Hospital, University of Cologne, 50937 Cologne, Germany; alina.kasdorf@uk-koeln.de (A.K.); raymond.voltz@uk-koeln.de (R.V.); julia.strupp@uk-koeln.de (J.S.); 2 Center for Health Services Research, Faculty of Medicine and University Hospital, University of Cologne, 50937 Cologne, Germany; 3 Center for Integrated Oncology Aachen Bonn Cologne Dusseldorf (CIO ABCD), Faculty of Medicine and University Hospital, University of Cologne, 50937 Cologne, Germany

**Keywords:** terminal care, quality of life, patient care, hospitals, feasibility studies

## Abstract

**Background/Objectives**: Early identification of terminally ill patients is crucial for enhancing care, patient and care partner satisfaction, and healthcare staff confidence in discussing disease trajectories. Yet, timely recognition remains challenging. To address this, we developed a minimally invasive intervention (MINI) for general hospital wards. We aimed to evaluate the MINI’s feasibility in facilitating an earlier identification of terminally ill patients and improving patient reported outcomes in a hospital setting. **Methods**: This prospective, two-arm pre-post intervention study at a university hospital evaluated the MINI alongside usual care. Patient-reported outcomes, including quality of life (SF-12), palliative care needs (IPOS), and functional status (ECOG), were collected at baseline and every three months over 12 months. Participants were allocated to a control or intervention group. **Results**: Of 188 patients identified using the Surprise Question, 58 completed the baseline assessment. While physical functioning (SF-12 PCS) remained comparable, the intervention group experienced clinically meaningful improvements in mental health (SF-12 MCS) at three months, with positive trends at six months. This group also showed a decline in palliative care needs, reduced emotional symptoms, and improved performance status, evidenced by significant differences in non-parametric analyses. These findings underscore the MINI’s potential to significantly improve patient well-being. **Conclusions**: This pilot study demonstrated the feasibility of the MINI and suggests it may foster meaningful system-wide change in patient-centred care within acute hospital settings, leading to improved patient outcomes and more confident healthcare staff in identifying terminally ill patients. However, given the small sample size, these findings should be interpreted with caution. Future research with larger cohorts and extended intervention periods is warranted to fully elucidate the MINI’s impact and refine strategies for improving care for terminally ill patients.

## 1. Introduction

Palliative care is often only associated with the dying phase, but its scope is broader than that [1,2,3]. It involves early planning and the consideration of the wishes and needs of patients and their family and friends regarding care options and their preferred place of death in their last year of life. It emphasizes enabling patients and their families to maintain the highest possible quality of life until the end of their lives [2].

On a global scale, the increasing prevalence of chronic, life-limiting conditions, including cancer, cardiovascular diseases, respiratory diseases, and renal diseases, as well as ageing populations, underscores the growing need for comprehensive palliative care interventions within healthcare systems [4]. Terminally ill patients in their last year of life face various imponderables [5]. Owing to the advanced and incurable nature of the disease, care provision creates multidimensional treatment and delivery requirements, all of which influence health-related quality of life and contribute to perceived suffering [6,7]. Although evidence supports the need for palliative care in both cancer and non-cancer patients, inequalities in communication and access to specialist palliative care persist [8]. Moreover, late and non-empathetic communication about the prospect of death is associated with distress, whereas empathic discussions are linked to fewer transitions in the last year of life [9]. Data suggest that 40.7% to 96.1% of patients who die would benefit from palliative care [10]. Early communication about the disease and care planning can improve quality of life, increase patient autonomy, and enhance satisfaction [11].

Nevertheless, identifying patients in their last year of life, particularly those with conditions other than cancer, remains challenging, leading to delays in palliative care referral, unmet palliative care needs, and difficulties in shared decision making about care options [12,13]. Consequently, patients’ wishes often remain unclear for too long [11]. Beyond patient outcomes, the quality of end-of-life care is profoundly influenced by the well-being and engagement of family and friend caregivers. Understanding and supporting caregivers by addressing aspects such as their quality of life [14], burden [15], self-efficacy, confidence, and preparedness [16,17] is an integral part of comprehensive palliative care.

General hospital wards serve as critical checkpoints in the last year of life. In a preceding study by our research team [5], 91% of terminally ill patients were hospitalized at least once during their last year of life. This setting thus provides an ideal opportunity to determine whether a patient is terminally ill and in need of palliative care and to initiate appropriate care accordingly. Unfortunately, this opportunity is rarely seized. In addition, satisfaction with general hospital services is the lowest among all healthcare settings, particularly compared with nursing homes and home care [6,13]. Although 68% of patients would have preferred to die at home, between 42% and 47% of deaths in Germany occur in hospitals [5]. In most cases, deaths in the last year of life are not unexpected, but result from previously diagnosed progressive diseases, the interaction of risk factors or diseases (multimorbidity), or the end of a natural ageing process. Particularly for non-cancer diseases, integration into palliative care structures may be delayed or absent [8]. Transitions from curative to palliative care are fluid. However, there is no consensus on the optimal timing of palliative care integration, although earlier involvement is thought to be most beneficial. Additional barriers include the life-sustaining culture of hospitals, where preventing death is the norm, combined with budgetary and time constraints and the absence of established standards for patients likely to die in the foreseeable future [18].

To improve care and satisfaction for terminally ill patients and their families while enhancing healthcare professionals’ (HCPs) confidence and competence, we developed a minimally invasive intervention (MINI) that incorporates simple yet effective clinical tools. This intervention employs a two-sided, trigger-question-based approach involving HCPs, patients, and family carers [6]. We aimed to evaluate the MINI’s feasibility in facilitating an earlier identification of terminally ill patients and improving patient-reported outcomes in a hospital setting. Specifically, using patient-reported outcome measures (PROMS), we investigated key healthcare outcomes, including quality of life, palliative care needs, and functional status in terminally ill patients. By promoting the early identification of palliative care needs and fostering communication through collaborative decision making, the MINI may address critical gaps in end-of-life care [2,12,19].

## 2. Materials and Methods

### 2.1. Study Design

This phase II pilot study is part of the broader project “Last Year of Life Study Cologne—Part 2 (LYOL-C-II)”. The study employs a prospective interventional design using a mixed-methods approach in a pre-post structure divided into two phases, as detailed in the study protocol [6]. This paper reports data collected from terminally ill patients during the second phase. The effects of the intervention on hospital staff (Kasdorf et al., in preparation [20]) and care partner data, including the Zarit Burden Interview [15] (Grimm et al., in preparation [21]), are reported separately. Patient data were used to assess the feasibility and potential of the minimally invasive intervention (MINI), alongside usual care, to enhance patient-reported outcomes, including quality of life (SF-12 [22]), palliative care needs (IPOS [23]), and functional status (ECOG [24,25]). The study was conducted in accordance with the Declaration of Helsinki and received ethical approval from the Ethics Commission of the Faculty of Medicine of Cologne University (20-1431). The trial is registered in the German Clinical Trials Register (DRKS00022378, registration date: 10 February 2021). This study was reported in accordance with the STROBE Statement for observational studies [26]. The STROBE checklist can be found in the Supplementary Material.

### 2.2. Intervention and Study Procedure

The intervention was developed to facilitate the early identification of patients in their last year of life and to prompt HCPs to revisit and revise the existing care plan. To this end, it integrates the *12-month Surprise Question (SQ)* (“Would I be surprised if this patient died in the next 12 months?”) [27] and the *Supportive and Palliative Care Indicators Tool* (SPICT-DE™) [28], both of which are concise, validated screening instruments designed to flag individuals who may benefit for palliative services and guide advanced care planning discussions. In addition, patients and their care partners received a “*Conversation Guide for Your Healthcare*,” a question prompt sheet (QPS) intended to structure and enable dialogues about prognosis, preferences, and care priorities for patients and their care partners [6].

**Figure 1:**
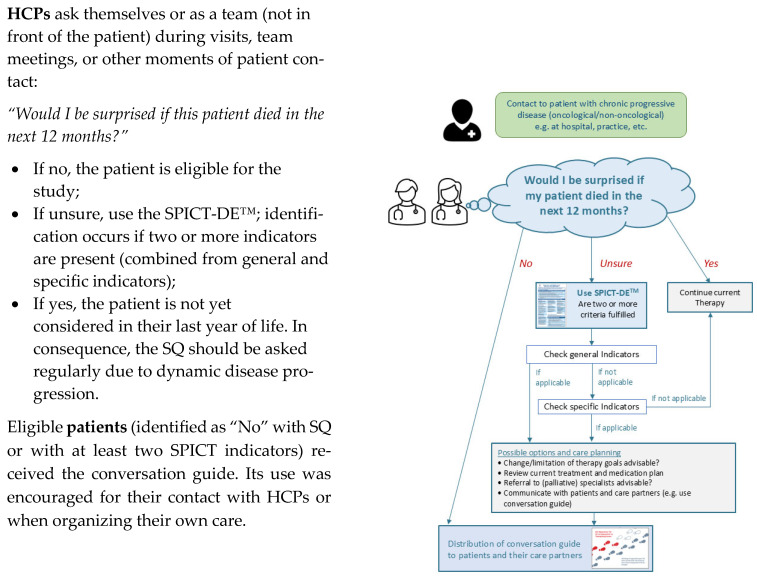
Illustration of the minimally invasive intervention (MINI).

Patients were allocated to either a control arm—receiving usual care and assessed prior to implementation of the MINI—or an intervention arm, in which usual care was supplemented by the MINI. In the control arm, a small team of senior physicians applied the 12-month SQ, either individually or collaboratively to identify eligible patients. In the intervention arm, all ward staff (physicians, nurses, and case managers) underwent a single comprehensive 60–90 min training session that addressed core palliative care principles, the rationale for early integration, and empathic communication techniques for discussing terminal illness. After the training, staff used the SQ in conjunction with SPICT-DE™ to screen all patients and encourage patients (and their care partners) to use the QPS during discussions about care preferences and trajectories. A schematic overview of the MINI is shown in Figure 1 (inspired by Afshar et al. [29]).

### 2.3. Recruitment Procedure

Recruitment for this prospective, two-arm pre-post intervention study followed a four-step procedure:

1. **Initial Screening**: HCPs (medical and nursing staff) on participating wards (Ward 1, primarily treating non-cancer patients; Ward 2, cancer inpatients/outpatients) identified eligible patients. This occurred during regular contact (visits and team meetings) or with study personnel (researchers (JG, BW) or study nurse (LW)) present at least twice weekly. **Inclusion criteria were** (a) patients who were identified via the Surprise Question (“Would you be surprised if this patient died within 12 months?” to which the answer was recorded as “no”) and the SPICT-DE™ tool; (b) aware of their incurable disease and limited prognosis; (c) aged ≥ 18 years; (d) capable of providing informed consent; (e) cognitively able to participate; and (f) fluent in German;
2. **Recruitment Log Completion**: For each flagged patient, HCPs completed a standardized recruitment log and confirmed all inclusion criteria, especially the patient’s awareness of their condition’s incurability;
3. **Patient Contact Form**: HCPs provided identified patients with a contact form to indicate their willingness to be contacted by the study team. These forms were securely collected by study personnel from the physician’s office. Study personnel regularly contacted wards to ensure ongoing screening;
4. **Informed Consent and Baseline**: Study personnel retrieved forms, contacted interested patients, and provided detailed study information. If patients were interested, the conversation guide (QPS) was distributed and their written informed consent was obtained. Care partner contact details were also collected for burden assessment, with this data to be published separately (Grimm et al., in preparation [21]). A baseline assessment (T0) was scheduled only after consent was secured.

### 2.4. Data Collection

The study, initially planned for 36 months starting May 2020, aimed to recruit 36 patients per group, totaling 72 participants [6]. Overall data collection spanned from February 2021 to August 2023. Recruitment for the control group (CG) in Ward 1 (non-cancer patients) started in February 2021, followed by the intervention group (IG) in April 2022. In Ward 2 (cancer inpatients/outpatients), CG recruitment began in June 2021, with IG recruitment in September 2022. Both concluded in August 2023.

Quantitative data were collected over 12 months for both CG and IG at five time points: baseline (T0) at enrolment, and at 3 (T1), 6 (T2), 9 (T3), and 12 months (T4) post-enrolment. Data were gathered through face-to-face interviews at the University Hospital, patients’ homes, or via telephone. Researchers read instructions and questions aloud, and participants responded verbally. Interviews typically lasted 40–90 min, varying with patient health and mood. In addition to quantitative data, field notes from HCPs, patients, and relatives, as well as interview comments, provided qualitative insights. Due to various implementation delays (discussed elsewhere), only T0 to T2 results will be compared for a clearer analysis of control and intervention phases.

### 2.5. Measurements

*Participant Characteristics.* We collected patients’ sociodemographic and specific disease characteristics at baseline (T0) and their preferred final place of care at each time point (T0–T4). Reasons for non-participation were also assessed.

*Potential effects*. The primary patient outcome was self-reported quality of life (QoL) using the “*Short-Form Health Service (SF-12)*” [22,30,31,32]. The SF-12 is commonly used for measuring QoL using two norm-based summary scales (*Physical Component Score*, PCS-12; *Mental Component Score*, MCS-12) with a population mean of 50 and a standard deviation of 10. The possible range for each score domain is 0–100, with higher scores indicating better QoL. Further, we assessed palliative care needs using the “*Integrated Palliative care Outcome Scale (IPOS)*”. It consists of a total sum scale (0–68) and three subscales (*physical symptoms* (0–40), *emotional problems* (0–16), and *communication and practical issues* (0–12)) [23]. The higher the score, the greater the need. Moreover, the functional status of the patients was assessed during the survey by the respective interviewers. For this purpose, the “*Eastern Cooperative Oncology Group Performance Status (ECOG*)” [24,25] was used with scores from 0 to 5, in which higher values indicate lower performance status.

### 2.6. Statistical Analysis

Data are presented as the mean ± standard deviation for continuous variables and as counts (percentages) for categorical variables. Group differences were assessed using independent samples t-tests for normally distributed data and Mann–Whitney U tests otherwise, with normality being assessed via the Shapiro–Wilk test. A significance threshold of *α* < 0.05 was used. No a priori dichotomization (“high” vs. “low”) was performed to preserve validity in this exploratory pilot study. The intervention effects were evaluated using linear mixed-effects models (LMMs) with restricted maximum likelihood (REML) estimation and Satterthwaite approximation for degrees of freedom. Time was modelled as a categorical factor with three levels (baseline, three months, and six months). Fixed effects included group, time, group × time interaction, gender, ward, and age (mean-centred), with categorical predictors being dummy-coded (reference: intervention group, six months, male, gynecology ward). Random intercepts were specified for each participant and an AR(1) residual covariance structure was applied. Random slopes and alternative structures were tested but were omitted due to convergence issues. Missing data in this pilot were documented at each time point and handled under the missing at random (MAR) assumption via likelihood-based REML to include participants with partial data. Due to the exploratory nature of the pilot study, formal multiplicity adjustment (e.g., Bonferroni corrections) was omitted to avoid obscuring potentially meaningful associations. To mitigate potential selection bias from screening, we used standardized screening logs. All analyses were conducted using SPSS version 28. Qualitative comments and field notes were analyzed following the approach outlined by Miles et al. [33].

## 3. Results

### 3.1. Allocation and Dropout

**Figure 2:**
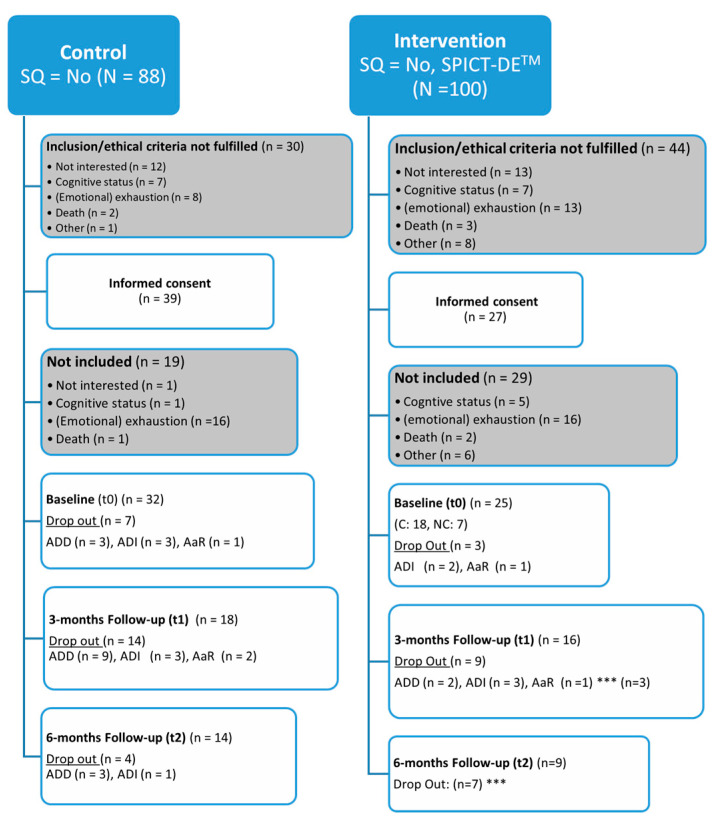
Flowchart with allocation and dropout details with classification of attrition based on Higginson et al. [34,35] (attrition due to death (ADD), attrition due to illness (ADI), attrition at random (AaR)); *** attrition due to end of study period.

A total of 188 patients were identified using the Surprise Question within the control and intervention phase. Of these, 110 met the formal inclusion criteria, of whom 66 signed the informed consent form. However, only 58 started the baseline assessment. Among these, 33 (56.9%) were control participants and 25 (43.1%) were intervention participants. This corresponds to an overall participation rate of 50.9%, with rates of 55.2% for the control group and 42.86% for the intervention group. In the control phase, 88 patients were identified using the SQ, with 49 patients on the non-cancer ward and 39 patients on the cancer ward. Despite more non-cancer patients being initially identified, slightly more cancer patients eventually participated. In the intervention phase, the distribution was comparable: 47 of the 100 identified patients were on the non-cancer ward, and 50 were on the cancer ward. Despite this balance, a higher proportion of cancer patients participated in the study (18 vs. 6). The non-participation of patients was attributed to various reasons: a lack of interest (33.8%), emotional or physical exhaustion (28.4%), cognitive impairment (18.9%), death (6.8%), and other reasons (12.2%). Figure 2 provides allocation and dropout details.

### 3.2. General Sample Characteristics at Baseline

A total of 79.3% of the patients were female and 20.7% were male. The proportion of women outnumbered men in both the control and intervention groups (75.8% and 84%, respectively). At baseline, the mean age of the patients was 63.28 years ± 13.43, with the mean age of the control patients being marginally higher (64.76 years ± 13.63 years) than that of the intervention patients (61.32 years ± 13.17 years). Male patients were on average approximately 10 years older. Gender differences in age were greater in the intervention group, with a mean age of 48 years for female intervention participants compared to 72 years for male intervention participants. A total of 67% of patients in the control group and 56% in the intervention had a nursing care grade. If patients had a nursing care grade, they were more likely to have a higher care grade, i.e., care grades 2–4, and, thus, were more dependent on help (Spearman’s *p* = 0.636, *p* < 0.001). The majority of patients stated that they had known about their underlying disease for at least one year or longer (82.5%). In the control group, 12.1% had known about their illness for at least one month but less than six months. In the intervention group, 8.3% stated that they had only learnt about their illness 24 h ago. Looking at the distribution of the underlying disease, more participants had cancer (60.3%). The patients consistently preferred their own home or a hospice as their final place of care. Approximately 21.4% of patients reported experiencing financial hardship due to their illness; however, a greater proportion of control patients reported this issue (31.3% vs. 8.3%). Details are shown in Table 1.

### 3.3. Self-Reported Health-Related Quality of Life with the Short-Form Health Service (SF-12)

#### 3.3.1. Physical Component Score

**Figure 3:**
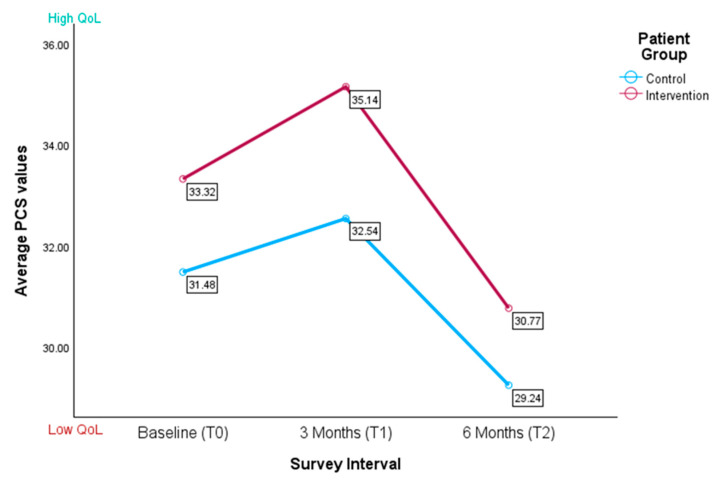
Average physical component scores for control and intervention groups (*n* = 58); the higher the score, the better the patient’s physical quality of life.

The mean physical quality of life of patients was found to be low (see Figure 3 and Table 2). Across both groups and at each time point, the data were found to be below the average German standard sample mean of 48.22 ± 8.77. At baseline (T0), the mean PCS for the intervention group was marginally higher (M = 33.40, SD = 5.50) in comparison to the control group (M = 31.34, SD = 5.53). However, these differences were not significant for the PCS between the intervention group and the control group (t(56) = −1.246, *p* = 0.109). Following a three-month period (T1), both groups demonstrated a slight increase in their scores, with the intervention group continuing to report higher scores (M = 35.14, SD = 7.20) in comparison to the control group (M = 32.54, SD = 6.51).

The differences were not significant (t(28.621) = −1.081, *p* = 0.142). At the six-month follow-up (T2), both groups demonstrated a decline in PCS, with the intervention group reporting an average score of M = 29.74 (SD = 4.93) and the control group exhibiting an average score of M = 29.24 (SD = 5.54). Comparable to the T0 and T1 results, these differences did not reach statistical significance (t(18) = −0.590, *p* = 0.281).

A linear mixed-effects model was used to compare the PCSs at baseline, three months, and six months between the control and intervention groups. The group × time interaction was not significant (F(2, 49.06) = 0.02, *p* = 0.977, see Table 2B), indicating no differential effects over time. There was a significant effect of time (F(2, 47.70) = 4.00, *p* = 0.025), with PCS being higher at baseline and three months than at six months. However, neither of these contrasts reached significance (β _T0 vs. T2_ = 2.64, *p* = 0.233; β _T1 vs. T2_ = 3.48, *p* = 0.111, see Table 2A). No significant main effect of group was found (F(1, 60.78) = 0.44, *p* = 0.511). Among the covariates, only ward was significant, with patients in the internal medicine ward scoring, on average, 4.45 points lower than those in the gynecology ward (F(1, 54.69) = 5.04, *p* = 0.029; β = –4.45, *p* = 0.029). The random intercept variance was 11.20 and residual variance was 20.44 (ICC ≈ 0.35). The AR(1) autocorrelation was low (ρ = 0.17, *p* = 0.692, see Table 2C).

#### 3.3.2. Mental Component Score (MCS)

**Figure 4:**
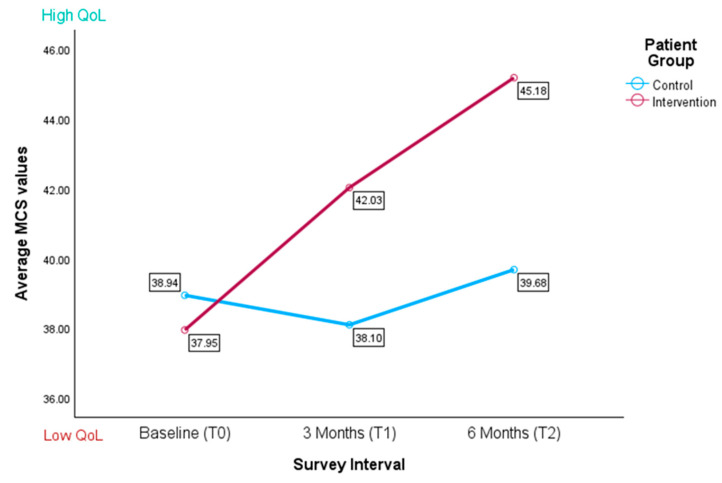
Average mental component scores for control and intervention groups (*n* = 58); the higher the score, the better the mental quality of life.

At baseline (T0), the control group (M = 38.63, SD = 7.53) and the intervention group (M = 38.09, SD = 7.25) showed no significant difference in MCS (t(56) = −0.287, *p* = 0.388). After three months (T1), the intervention group (M = 42.03, SD = 4.82) reported significantly higher MCSs than the control group, indicating a better QoL (M = 38.10, SD = 7.06, t(29.691) = −1.894, *p* = 0.034). At six months (T2), the intervention group continued to report higher MCSs (M = 44.67, SD = 6.24) compared to the control group (M = 39.68, SD = 7.27), although the difference was not statistically significant (t(18) = −1.525, *p* = 0.071). Both groups showed below-average MCSs compared to the German standard sample mean (for 1998) of 51.41 ± 8.55 (see Figure 4).

An LMM compared MCSs between the control and intervention groups at baseline, three months, and six months. The group × time interaction was not significant (F(2, 55.34) = 2.00, *p* = 0.146, see Table 3B), indicating no difference between the groups over time. The main effect of time was also not significant (F(2, 54.25) = 2.24, *p* = 0.116), although baseline scores were lower than 6-month scores (β _T0 vs. T2_ = –6.21, *p* = 0.032, see Table 3A). There were also no significant main effects of group (F(1, 51.64) = 1.58, *p* = 0.215) and none of the covariates were significant (all *p* > 0.05). The random intercept variance was 19.04 and the residual variance was 31.88 (ICC ≈ 0.37). The AR(1) autocorrelation was non-significant (ρ = –0.20, *p* = 0.433, see Table 3C). 

### 3.4. Palliative Care Needs Using the Integrated Palliative Care Outcome Scale (IPOS)

#### 3.4.1. IPOS Number of Physical, Emotional, and Communication Problems

At baseline, both the control and intervention patients reported on average having 11 out of 17 possible problems, encompassing physical or emotional symptoms as well as communication or practical issues (control: SD = 2.59, range = 6–14; intervention: SD = 2.74, range = 5–15). Over time, the average number of reported problems decreased for both groups, although no significant differences were observed.

#### 3.4.2. IPOS Total Scores

**Figure 5:**
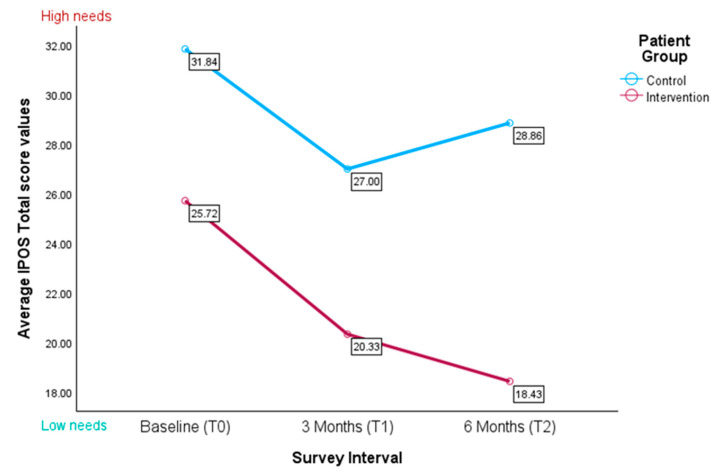
Average IPOS total scores for the control and intervention group (*n* = 58); the higher the score, the higher potential palliative care needs.

The IPOS scores for the control group initially demonstrated a decline, followed by an upward trajectory. In contrast, the intervention group exhibited a pronounced downward trend, indicating better symptom management, especially from the baseline to the three-month follow-up, as seen in Figure 5.

The Mann–Whitney U tests revealed that, at all three time points, the control patients had significantly higher total sum scores than the intervention patients, indicating greater palliative care needs in the control group (t0: U = 250.000, Z = −2.415, *p* = 0.008; t1: U = 83.500, Z = −1.865, *p* = 0.031, t2: U = 21.500, Z = −2.055, *p* = 0.020). In particular, the IPOS scores for the control group initially declined and subsequently increased, whereas the intervention group exhibited a pronounced downward trend—most notably from the baseline to the three-month follow-up (see Figure 5). 

An LMM revealed no significant group × time interaction (F(2, 47.68) = 0.45, *p* = 0.638, see Table A2) or main effect of time (F(2, 46.74) = 1.91, *p* = 0.159). However, there was a significant main effect of group (F(1, 56.79) = 6.43, *p* = 0.014), with control patients scoring on average 8.8 points higher than the intervention group (β = 8.77, *p* = 0.030). None of the covariates were significant (all *p* > 0.05), though there was a positive trend for age (*p* = 0.068). Variance component estimates indicated modest between-subject variability (random intercept variance = 8.89, residual variance = 85.90, ICC ≈ 0.09) and non-significant AR(1) autocorrelation (ρ = 0.62, *p* = 0.113).

#### 3.4.3. IPOS Subscale for Physical Symptoms

**Figure 6:**
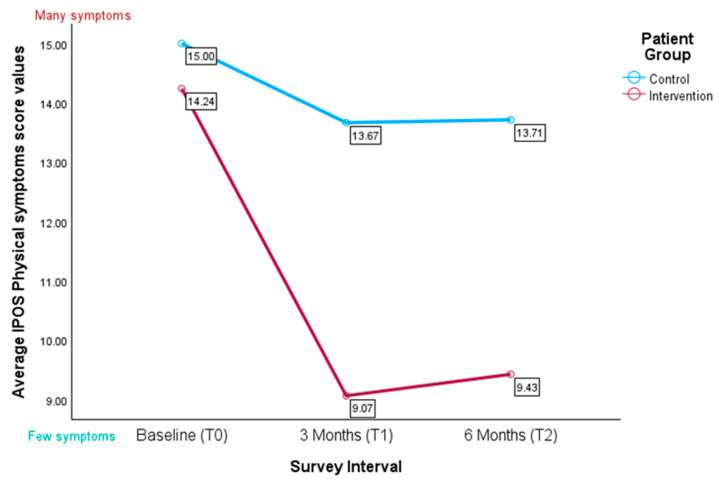
Average values for the IPOS subscale for physical symptoms (*n* = 58); the higher the score, the higher perceived physical symptom burden.

The Mann–Whitney U test revealed a significant difference at three months, with the control group reporting higher physical symptom scores (U = 83.000, Z = −1.886, *p* = 0.030; see Figure 6). A linear mixed-effect model showed a trend for the group × time interaction towards significance (F(2, 39.82) = 3.13, *p* = 0.055; see Table 4B). There was no main effect of time (F(2, 39.19) = 0.98, *p* = 0.384). Pairwise contrasts relative to the six months reference indicated higher scores at baseline (β = 3.75, *p* = 0.071) and at three months (β = 0.60, *p* = 0.747; see Table 4A), neither of which reached significance. The omnibus test for group was marginal (F(1, 55.48) = 3.43, *p* = 0.069), but the specific group contrast was significant, with control patients scoring 5.71 points higher on average than intervention patients (β = 5.71, *p* = 0.039). None of the covariates were significant (all *p* > 0.05), although age showed a trend towards significance (*p* = 0.085). Variance component estimates indicated substantial between-subject variability (random intercept variance = 29.31, residual variance = 21.62, ICC ≈ 0.58). The AR(1) autocorrelation was non-significant (ρ = 0.38, *p* = 0.381; see Table 4C). 

#### 3.4.4. IPOS Subscale for Emotional Symptoms

**Figure 7:**
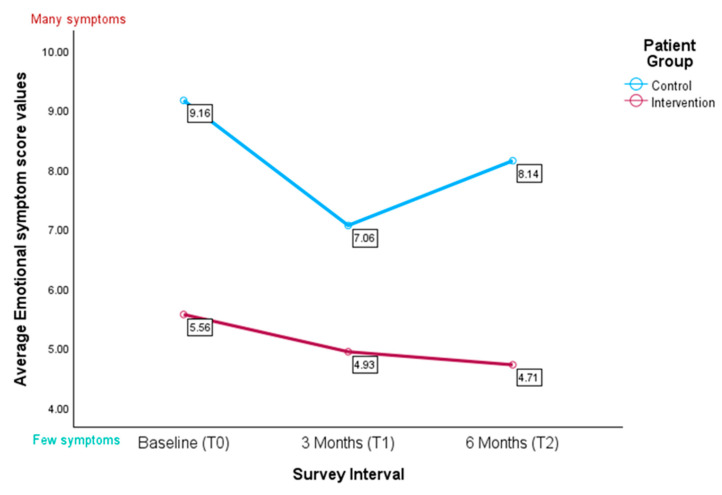
Average values for the IPOS subscale for emotional symptoms (*n* = 58); the higher the score, the higher perceived emotional symptom burden.

The control group showed higher mean scores for the subscale for emotional symptoms compared to those in the intervention group. These differences were statistically significant at baseline (U = 182.000, z = −3.525, *p* < 0.001) and at six-month follow-up (U = 19.000, z = −2.257, *p* = 0.023) (see Figure 7). 

A linear mixed model revealed no significant group × time interaction (F(2, 55.92) = 0.78, *p* = 0.466, see Table 5B). There was also no main effect of time (F(2, 54.31) = 1.33, *p* = 0.272). The omnibus test revealed a significant main effect for group (F(1, 49.20) = 10.44, *p* = 0.002), though the specific contrast trended towards significance only (β = 2.76, *p* = 0.075, see Table 5A). None of the covariates were significant (all *p* > 0.05). Variance component estimates (Table 5C) showed a residual variance of 11.29 and a random-intercept variance of 0.37 (ICC ≈ 0.03). The AR(1) autocorrelation was non-significant (ρ = 0.37, *p* = 0.387).

The qualitative data (field notes) also revealed emotional concerns held by the patients. In particular, some patients expressed the wish to not think about their illness all the time. One patient mentioned the following:

*“I don’t want to have anything else besides therapy. Otherwise, it’s too much. I’d like to be able to stop thinking about my illness.” (Pat_KG_FC4)*.

(extracted as free text comments)

#### 3.4.5. IPOS Subscale for Communication and Practical Issues

In the subscale pertaining to communication and practical issues, the control patients exhibited slightly higher average scores at baseline in comparison to the intervention patients (M = 5.781 vs. M = 4.160), suggesting more prevalent issues in the control group within this domain. The observed difference at baseline was statistically significant (U = 259.000, Z = −2.303, *p* = 0.010).

At three months (T1), the intervention patients showed an increase in their scores (M = 4.889), indicating a greater need in this area, while control patients exhibited a decrease (M = 5.133). However, the observed difference at T1 was not statistically significant (U = 134.500, Z = −0.018, *p* = 0.497).

**Figure 8:**
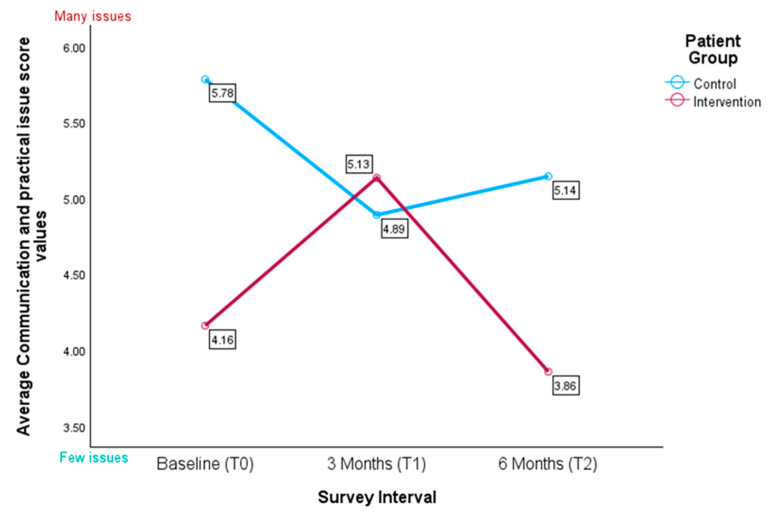
Average scores for the IPOS subscale for communication and practical issues (*n* = 58); the higher the score, the more perceived communication and practical issues.

By six months (T2), the intervention patients demonstrated a notable decrease in reported communication and practical issues (M = 3.857), whereas the control patients’ scores remained relatively stable (M = 5.143). The difference at T2 was statistically significant, with lower scores indicating fewer issues in the intervention group compared to the control group (U = 24.500, Z = −1.870, *p* = 0.029) (see Figure 8).

To further examine these differences while accounting for potential confounders, we conducted a linear mixed-effects model. The LMM did not reveal a significant group × time interaction (F(2, 57.67) = 2.44, *p* = 0.096). There were also no significant main effects of group (F(1, 42.60) = 2.08, *p* = 0.157) or time (F(2, 56.31) = 0.47, *p* = 0.626). None of the covariates were significant (all *p* > 0.05). Variance component estimates showed a residual variance of 4.20 and a random intercept variance of 0.75 (ICC ≈ 0.15). The AR(1) autocorrelation was non-significant (ρ = 0.06, *p* = 0.872; see Table A3).

The qualitative data revealed the communication challenges experienced by patients, aligning with the quantitative findings. In particular, some patients expressed difficulties in discussing their end-of-life care preferences and concerns with their families. For example, one patient shared the following:

*“Can I do that to my wife, having her care for me at home until the end? I would rather die at home than in a nursing home or something like that, but I don’t even dare to think about it or talk to her about it just yet”* (PAT_KG_CP15).

(extracted as free text comments)

Another key concern raised by the patients was the need for continuous care from a fixed contact person to discuss problems and receive necessary information. A patient described their experience as follows:

*“Although I’ve tried to discuss my worries and fears, it hasn’t worked because I always see someone else. The main contact person is good, but you hardly see them as a patient because they have so little time. The constant change of doctors makes you feel like a number, so you can’t really talk about your fears.” [...] (PAT_IG_KR4)*.

(extracted as free text comments)

### 3.5. Cooperative Oncology Group (ECOG) Performance Status

**Figure 9:**
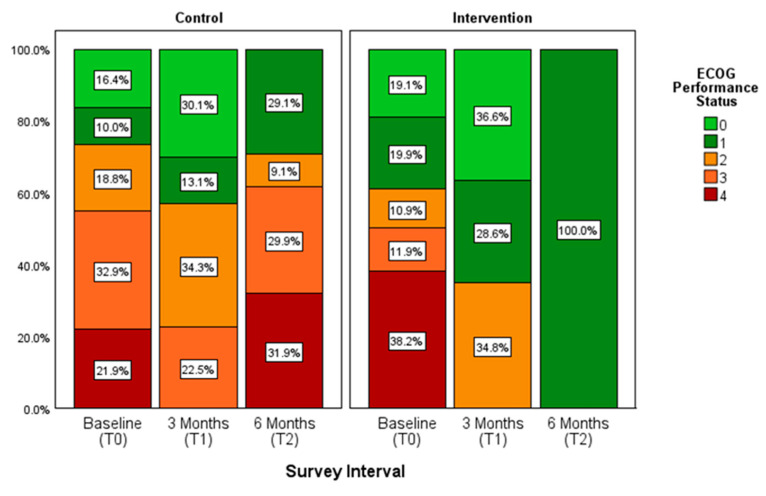
Average functional status of control and intervention group (*n* = 58); the higher the performance status (0–4), the lower the functional status.

The data indicate that, on average, patients in the control group had poorer performance scores and were potentially more reliant on assistance from others. While the mean ECOG scores of the intervention patients showed a slight decline, those of the control patients remained relatively stable between T1 and T2. A significant difference was observed between the control and intervention mean scores at three months (U = 77.000, Z = −2.234, *p* = 0.011) and six months (U = 21.000, Z = −2.054, *p* = 0.044). See Figure 9.

In addition, a linear mixed model revealed no significant group × time interaction (F(2, 44.48) = 1.32, *p* = 0.278; see Table 6B) or time effect (F(2, 44.00) = 0.95, *p* = 0.394). There was a trend towards a main effect of group (F(1, 56.03) = 3.04, *p* = 0.087). The specific group contrasts were significant (β = 0.78, *p* = 0.040; see Table 6A), with control patients scoring 0.78 points higher (i.e., worse PS) than intervention patients. Among the covariates, only ward was significant (β = 1.05, *p* = 0.004), indicating poorer PS in the internal medicine ward. Variance components revealed substantial between-patient variability (residual variance = 0.32, random intercept variance = 0.54, ICC ≈ 0.63) and a non-significant AR(1) autocorrelation (ρ = 0.19, *p* = 0.561; Table 6C).

## 4. Discussion

Our study evaluated the feasibility of implementing the MINI in two general university hospital wards with the primary objective of facilitating an earlier identification of terminally ill patients. This aimed to improve patient outcomes and foster sensitive, early communication regarding disease. We hypothesized that the intervention would enhance patient-reported outcomes, and our findings suggest a discernible positive trend supporting this. The intervention group demonstrated a greater improvement in their mental quality of life and generally exhibited a superior ECOG performance status. Conversely, the control group exhibited higher total IPOS scores, indicating a greater symptom burden, particularly in the physical and emotional domains. Although the intervention group initially reported more communication and practical issues, a marked improvement was observed by the six-month mark, suggesting potential long-term benefits even if short-term gains were less pronounced.

### 4.1. Allocation and General Sample Characteristics

In the control group, only senior physicians were asked the Surprise Question (SQ), whereas in the intervention group, all medical and nursing staff were instructed to do so. Additionally, the intervention phase introduced SPICT-DE^TM^ and an information hour, aiming to involve more staff and increase their awareness of palliative needs. These changes likely contributed to baseline differences between the two groups.

Identifying limited life expectancy appeared to be more straightforward for cancer patients, reflected in the intervention group’s higher identification rate among this population. However, inviting more severely burdened patients also led to more individuals being too ill or dying before giving consent, especially in the non-cancer setting. Contrary to initial assumptions, broadening responsibility sometimes reduced ownership: recruitment declined once senior physicians were less involved. This “diffusion of responsibility” [36] highlights the importance of a designated lead, such as a senior physician, to effectively distribute responsibility. A higher proportion of female patients participated in both phases, largely due to recruitment from a gynecological ward. We deliberately included both a non-cancer ward and a cancer ward to address known gaps in early palliative identification, which is often pronounced in oncology [37,38] and among patients with chronic diseases. By covering both wards, we aimed to strengthen healthcare outputs for both cancer and non-cancer patients and expose systemic barriers to timely referral.

Finally, a greater proportion of patients in the intervention group reported having earlier knowledge of their condition’s incurability. The MINI’s systematic screening and proactive communication likely encouraged staff to speak directly with patients about their health status, aligning with studies indicating that training and a supportive environment facilitate earlier prognostic disclosure [39,40]. Moreover, the hospital context may have normalized these conversations, as staff were urged to identify terminally ill patients and address their diagnosis [41], and admission itself can serve as an opportunity to identify patients approaching the end of their life [5]. However, factors beyond the MINI, such as ward culture, physician communication styles, and individual patient circumstances, also affect when patients learn of their condition’s incurability.

### 4.2. Outcome Measures

#### 4.2.1. SF-12

In both groups, PCSs were consistently below the German standard mean (48.22 ± 8.77), reflecting their advanced disease status and the progressive nature of their disease. Neither the intervention nor the control group showed a distinct advantage in PCS at any time point, suggesting that, within this sample, the intervention did not confer a measurable benefit in terms of physical functioning over and above usual care. However, the initial improvement from T0 to T1 may highlight a short-term positive effect—whether from increased attention, symptom management, or other supportive measures. Disease progression likely overshadowed any gains in physical functioning over time, highlighting the need for robust symptom management and rehabilitation strategies alongside psychosocial interventions [42,43,44]. In contrast to the PCS, the MCS results showed more promising trends. In independent samples t-tests, the intervention group’s MCS increased significantly from baseline to three months and remained higher at six months, though this latter difference did not reach statistical significance. However, in the mixed-model analysis, no significant treatment effect emerged, likely due to the small sample size and limited statistical power. Nevertheless, the MINI’s emphasis on earlier identification and communication about palliative needs may have bolstered patients’ sense of control, reduced anxiety, and improved coping, even if these benefits were not fully captured. Contrary to common concerns, talking about problems and illnesses and planning care can improve QoL [11]. Engaging both medical and nursing staff likely offered emotional and informational support, dispelling misconceptions and improving the overall mental well-being of patients and their caregivers. Overall, these findings are in line with other studies and suggest that while physical quality of life may remain relatively stable or even decline in the face of advancing illness, mental quality of life can be positively influenced by interventions that focus on issues beyond the patient’s physical status and emphasize communication, emotional support, and early palliative care planning [43,45].

#### 4.2.2. IPOS

The IPOS provides a comprehensive view of patients’ palliative care needs including physical, emotional, and communication issues. Both groups reported multiple problems at baseline, reflecting the high burden of terminal illnesses. The results indicate that control patients consistently exhibited higher palliative care needs, as reflected in significantly higher IPOS scores at all time points. This aligns with previous findings that terminally ill individuals often experience a high symptom burden and unmet palliative care needs, not only due to physical symptoms but also because of the emotional distress associated with illness and its management [2,13]. Our findings reinforce the importance of structured palliative care approaches to address the complex needs of terminally ill patients. A modest effect on physical symptoms was observed. While the overall symptom burden tended to increase in both groups, the intervention group showed a less pronounced rise, consistent with the Mann–Whitney U test results at three months. In the mixed-model analysis, the group × time interaction trended towards significance, and the specific contrast indicated controls scoring higher on average. Although the small sample size limits definitive conclusions, these results indicate that the MINI may help mitigate the progression of physical symptoms in terminally ill patients.

We found that patients in the intervention group consistently reported fewer emotional symptoms than controls, with significant differences at baseline and six months, suggesting the MINI may alleviate emotional distress. The baseline advantage likely reflects the benefit of the staff training delivered to intervention personnel as part of the MINI. The mixed-model analysis confirmed a significant omnibus group effect, although the specific control vs. intervention contrast trended toward significance. Qualitative data further underscores patients’ struggle with emotional burdens and their wish to avoid constant rumination about their illness. Together, these findings suggest that interventions such as the MINI may help meet these needs and highlights the value of structured psychosocial support [46], potentially enabled by earlier and more frequent conversations facilitated by the MINI. However, further research is required to confirm its long-term impact.

Although the data showed a modest increase in communication scores for the intervention at three months, by six months, they had decreased significantly below the control group’s levels. This pattern suggests that while patients might initially feel overwhelmed by receiving a life-threatening diagnosis or discussions about care planning, over time, sustained engagement and communication pathways can help reduce barriers in communication and practical support and thus potentially increase QoL [47]. However, the linear mixed model did not reveal significant effects, suggesting that other factors may have influenced these results. Taken together, the IPOS findings suggest that targeted, proactive care planning may help mitigate certain palliative care needs [11].

#### 4.2.3. ECOG

Performance status (PS) is crucial for terminally ill patients, particularly those with cancer, as patients with a greater PS are less likely to experience adverse events and have a greater likelihood of responding well to treatment [48]. Although PS often declines over time, patients in the intervention group exhibited a slight improvement or stabilization in ECOG scores compared to the control group at both three and six months. This pattern may suggest that the intervention has helped maintain PS. This could be attributed to structured support, including symptom monitoring, improved coping, and heightened disease awareness [49]. However, the linear mixed model only trended toward a significant main effect of group, despite the specific contrast showing an advantage for the intervention arm. Moreover, the significant ward effect suggest that site-specific factors may have also influenced PS. Taken together, these results suggest a potential positive impact of the intervention on preserving functional status, which is important because a greater PS could also enable patients to tolerate treatments more effectively, underscoring the importance of early palliative support that addresses both physical and psychosocial factors [50].

### 4.3. Strengths, Limitations, and Lessons Learned

Our study’s prospective, mixed-method design provided direct, unbiased insights from terminally ill patients during the last year of their life, thereby overcoming the recall bias typical of retrospective studies. A further strength of this study is the diverse sample recruited, which included both cancer and non-cancer patients, alongside the active involvement of medical and nursing staff across multiple general hospital wards. This approach substantially enhances the real-world applicability of our findings.

Despite these strengths, our challenges included the limited sample size, data completeness, and the full implementation of the MINI. These challenges existed at individual (e.g., patient, family, and HCP), trial (e.g., recruitment barriers and workflow disruptions), and systemic (e.g., societal knowledge) levels.

A major limitation was gatekeeping by HCPs, a well-documented phenomenon in palliative care [51] often driven by concerns about patient distress and burden [52,53]. Staff sometimes explicitly discouraged referrals even when patients met the inclusion criteria. This reluctance aligns with findings that practitioners can experience intense emotional distress when breaking bad news, acting as a barrier to recruitment. Such concerns were exacerbated by limited time, resources, and training, with some HCPs worried that such conversations elicit physical and emotional responses, requiring more time and attention than they could realistically provide [39].

Furthermore, reluctance to use the SQ was observed. In the control phase, medical staff, particularly residents, often hesitated to answer ‘no’ for younger cancer patients or communicate the diagnosis’ implications. For instance, a resident’s decision to overrule a nurse’s suggestion, despite the patient’s metastases (“she was still so young…”) and the nurse’s reaction (“Are you saying this with your heart or with your mind?”), highlights this reluctance. Similarly, nurses, particularly in non-cancer wards, showed reluctance to use the SQ. This aligns with the literature noting prognostic issues as a substantial challenge for nurses, in which prognostication power is often still attributed solely to doctors [54,55]. This underscores the critical need for enhanced training to equip staff with confidence in identifying patients and discussing palliative care. Misconceptions that palliative care means immediate death, diminishing hope [3,56], or that offering non-curative treatment options signifies personal failure [57] further hindered SQ use and diagnosis discussions.

Data protection constraints also impacted recruitment. We could only recruit patients confirmed by their medical team as aware of their terminal illness. This is problematic, as HCPs’ perception of prognosis discussions may differ from patients’ and carers’ perceptions [58]. Consequently, some patients declined to participate due to unawareness of their terminal illness, believing the study was inappropriate (e.g., one patient stated she had surgery recently and thus did not meet the criteria for being terminal ill).

Patient health status [59] significantly impacted consent and retention. Non-cancer inpatients, often with more advanced conditions [60], exhibited high dropout rates before the baseline assessment or within the first three months, frequently due to death or rapid health deterioration. This instability was observed in both non-cancer and cancer inpatients compared to cancer outpatients. Even relatively stable outpatient cancer patients frequently declined to participate, consistent with Walshe et al. [53]. Terminally ill patients experience significant emotional and organizational burden from their treatment, fostering “double awareness” [61]—a simultaneous need for hope and intermittent reprieves from the reality of their illness. This made many reluctant to commit to our four quarterly interviews, leading to withdrawals driven by study duration, workload, negative associations with palliative care, and uncertainty about future health status. External disruptions—including the COVID-19 pandemic, a prolonged nurses’ strike (1 May 2022–20 July 2022), and the end of the funding phase (31 April 2023/31 October 2023)—prematurely curtailed the follow-up and impeded longitudinal data collection.

From the insights gained through this study’s implementation, several actionable recommendations emerge for optimizing the implementation of future palliative care interventions. Specifically, fostering a clear sense of ownership among all healthcare professionals is paramount. This can be achieved through the precise delineation of roles and a continuous emphasis on the value of early palliative identification in routine clinical practice. Furthermore, comprehensive and ongoing training is essential. This training should systematically address emotional challenges, correct misconceptions about palliative care, and build confidence in using screening tools effectively and facilitating sensitive end-of-life conversations. To mitigate patient burden, particularly in data collection, strategies such as flexible follow-up schedules or the adoption of alternative, less intrusive methods (e.g., shorter interviews or remote surveys) are recommended. Optimizing recruitment procedures necessitates ensuring patients have a profound understanding of the study’s aims, which can be achieved through its introduction by a familiar face they trust, and attention to appropriate researcher attire [62]. Finally, the establishment of robust contingency plans is critical to safeguard study continuity and resilience against unforeseen external disruptive events, such as pandemics or strikes. Adherence to these targeted strategies will facilitate a more effective integration of the MINI into existing hospital workflows, thereby fostering improved end-of-life care outcomes.

## 5. Conclusions

This pilot study indicates that system-wide change requires time and perseverance, but that the MINI can be a first step to meaningful change in healthcare. However, our findings must be interpreted with caution due to the inherently small sample size of this pilot study, even when the results appear significant. Notably, the intervention’s full impact may take longer to manifest, underscoring the importance of extended intervention periods, staff training on comprehensive communication, and adaptive recruitment strategies in future trials. Future studies with larger cohorts should build on these preliminary findings, refining the MINI approach and avoiding the pitfalls identified here. Considering the specific context of our study within a single university hospital and with particular patient characteristics, the broader applicability of these findings needs careful consideration. By doing so, we can increase our understanding on how disease trajectory, patient preferences, and staff training collectively influence outcomes, paving the way for future research that can continue moving from “mini” interventions to create truly meaningful changes in palliative care.

## Figures and Tables

**Table 1:** Sample characteristics of both control and intervention patients (*n* = 58).

Characteristics	Control n (%) M (SD)	Intervention n (%) M (SD)	Total n (%) M (SD)
Care partner enrolled in study			
No	18 (52.9)	19 (73.1)	37 (61.7)
Yes	16 (47.1)	7 (26.9)	23 (38.3)
Care setting of recruitment			
Internal medicine unit	16 (48.5)	7 (28.0)	23 (39.7)
Gynecological inpatient unit	4 (12.1)	2 (8.0)	6 (10.3)
Gynecological outpatient unit	13 (39.4)	16 (64.0)	30 (50.0)
Highest school qualification *			
High school or lower	12 (36.4)	10 (43.5)	22 (39.3)
Vocational degree	14 (42.4)	7 (30.4)	21 (37.5)
University degree	7 (21.2)	6 (26.1)	13 (23.2)
Age	64.76 ± 13.63	61.32 ± 13.17	63.28 ± 13.43
Sex			
Female	25 (75.8)	21 (84.0)	46 (79.3)
Male	8 (24.2)	4 (16.0)	12 (20.7)
Relationship status			
Single	2 (6.1)	7 (29.2)	9 (15.8)
Married/civil partnership	19 (57.6)	12 (50.0)	31 (53.6)
Divorced	7 (21.2)	2 (8.3)	9 (15.8)
Widowed	5 (15.2)	3 (12.5)	8 (14.0)
Religion (NA 2)			
None	6 (18.8)	6 (25.0)	12 (21.4)
Christianity	24 (75.0)	18 (75.0)	42 (75.0)
Islam	2 (6.3)	0 (0.0)	2 (3.6)
Employment			
No	25 (78.1)	16 (80.7)	40 (71.4)
Currently on sick leave	3 (9.4)	4 (16.7)	7 (12.5)
Yes	4 (12.5)	4 (16.7)	8 (14.3)
Children (NA 1)			
No	4 (12.1)	8 (33.3)	12 (21.1)
Yes	29 (87.9)	16 (66.7)	45 (78.9)
1	10 (31.3)	4 (16.7)	14 (25.0)
2	10 (31.1)	9 (37.5)	19 (33.9)
3 or more	8 (25.0)	3 (12.5)	11 (19.6)
Living situation (NA 1)			
Living alone	6 (18.2)	9 (37.5)	15 (26.3)
Living with another person	27 (81.8.4)	15 (62.5)	42 (73.7)
Care Grade ** (NA 1)			
No	10 (30.3)	11 (45.8)	21 (36.8)
Yes	23 (69.7)	13 (54.2)	36 (63.2)
Care grade 1	1 (3.0)	1 (4.2)	2 (3.5)
Care grade 2	8 (24.2)	2 (8.3)	10 (17.5)
Care grade 3	11 (33.3)	5 (20.8)	16 (28.1)
Care grade 4	3 (9.1)	4 (16.7)	7 (12.3)
Unsure	0 (0.0)	1 (4.2)	1 (1.8)
Planned/applied for	2 (6.1)	0 (0.0)	2 (3.5)
Knowledge about incurability (NA 3)			
<24 h	0 (0.0)	2 (8.7)	2 (3.6)
1 week to <1 month	0 (0.0)	1 (4.3)	1 (1.8)
1 to <6 months	4 (12.5)	1 (4.3)	5 (9.1)
6 months to <1 year	1 (3.1)	0 (0.0)	1 (1.8)
≥1 year	27 (84.4)	19 (82.6)	47 (86.6)
Financial problems			
No	16 (50.0)	14 (58.3)	30 (51.7)
Rather not	2 (6.3)	4 (16.7)	6 (10.3)
Rather yes	4 (12.5)	3 (12.5)	7 (12.1)
Yes	10 (31.3)	2 (8.3)	12 (21.4)
Not sure	0 (0.0)	1 (4.2)	1 (1.8)

Note: NA = no data; M = mean; SD = standard deviation. * *School qualification* based on German system. ** Nursing *care grades* are used in the German care system to classify people’s dependency on care as follows: 1. slight impairment of independence; 2. considerable impairment of independence; 3. severe impairment of independence; 4. most severe impairment of independence; 5. most severe impairment of independence with special requirements for nursing care.

**Table 2:** Mixed-effects model results for the SF-12 physical component score (PCS).

**A. Fixed-Effect Estimates**						
**Parameter**	**β**	**Std. Error**	**df**	**t-Value**	***p*-Value**	**95% CI**
Intercept	35.53	2.98	86.82	11.26	<0.001	[27.61, 39.44]
Group (control)	–1.16	2.55	79.50	–0.46	0.650	[–6.23, 3.91]
Time (T0 vs. T2)	2.64	2.17	30.96	1.22	0.233	[–1.79, 7.08]
Time (T1 vs. T2)	3.48	2.15	60.86	1.62	0.111	[–1.82, 7.79]
Group × Time (control × T0)	0.09	2.64	28.10	0.03	0.974	[–5.32, 5.49]
Group × Time (control × T1)	0.45	2.62	59.06	0.17	0.865	[–4.80, 5.69]
Gender (female)	–1.88	2.16	55.28	–0.87	0.387	[–6.20, 2.44]
Ward (internal medicine)	–4.45	1.98	54.69	–2.24	0.029 *	[–8.43, –0.48]
Age	−0.03	0.06	45.33	−0.42	0.675	[–0.15, 0.10]
**B. Type III Tests of Fixed Effects**						
**Parameters**	**Num df**	**Den df**	**F**	***p*-value**		
Group	1	60.78	0.44	0.511		
Time	2	47.70	4.00	0.025 *		
Group × Time	2	49.06	0.02	0.977		
Gender	1	55.28	0.76	0.387		
Ward	1	54.69	5.04	0.029 *		
Age	1	45.33	0.18	0.675		
**C. Variance Components and AR (1) Parameter**						
**Component**	**Estimate**	**Std. Error**	**95% CI**			
Random-intercept variance	11.20	11.70	[1.44, 86.85]			
Residual variance	20.44	10.77	[7.28, 57.38]			
AR(1)	0.17	0.44	[–0.61, 0.79]			

Note CI = confidence interval; df = degrees of freedom; β = unstandardized coefficient. * *p* < 0.05. Reference categories: Group = intervention; Time = T2 6 months; Gender = male; Ward = gynecology.

**Table 3:** Mixed-effects model results for the SF-12 mental component score (PCS).

**A. Fixed-Effect Estimates**						
**Parameter**	**β**	**Std. Error**	**df**	**t-Value**	**Sig.**	**95% CI**
Intercept	43.97	3.75	86.39	11.72	<0.001	[36.51, 51.42]
Group (control)	−4.49	3.28	79.41	−1.37	0.175	[−11.01, 2.04]
Time (T0 vs. T2)	−6.21	2.79	39.95	−2.23	0.032 *	[−11.85, 0.57]
Time (T1 vs. T2)	−2.45	3.04	56.84	−0.81	0.424	[−8.55, 3.64]
Group × Time (control × T0)	5.52	3.71	38.02	1.64	0.110	[−1.30, 12.34]
Group × Time (control × T1)	1.18	3.76	53.67	0.32	0.754	[−6.36, 8.72]
Gender (female)	0.47	2.64	53.38	0.18	0.859	[−4.82, 5.76]
Ward (internal medicine)	−0.52	2.41	51.50	−0.22	0.830	[−5.36, 4.32]
Age	0.037	0.07	39.47	0.51	0.611	[−0.12–0.18]
**B. Type III Tests of Fixed Effects**						
**Parameters**	**Num df**	**Den df**	**F**	***p*-value**		
Group	1	51.64	1.58	0.215		
Time	2	54.25	2.24	0.116		
Group × Time	2	55.34	2.00	0.146		
Gender	1	53.38	0.03	0.859		
Ward	1	51.50	0.05	0.830		
Age	1	39.47	0.26	0.611		
**C. Variance Components and AR (1) Parameter**						
**Component**	**Estimate**	**Std. Error**	**95% CI**			
Random-intercept variance	19.04	7.41	[7.71, 47.03]			
Residual variance	31.88	7.41	[20.21, 50.27]			
AR(1)	−0.20	0.26	[−0.62, 0.31]			

Note CI = confidence interval; df = degrees of freedom; β = unstandardized coefficient; * *p* < 0.05. Reference categories: Group = intervention; Time = T2 6 months; Gender = male; Ward = gynecology.

**Table 4:** Mixed-effects model results for the IPOS physical symptoms subscale.

**Pannel A. Fixed-Effect Estimates**						
**Parameter**	**β**	**Std. Error**	**df**	**t-Value**	**Sig.**	**95% CI**
Intercept	9.06	3.65	67.61	2.48	0.016 *	[−1.77, 16.34]
Group (control)	5.71	2.72	85.11	2.10	0.039 *	[0.31, 11.12]
Time (T0 vs. T2)	3.75	2.00	29.96	2.87	0.071	[−0.34, 7.84]
Time (T1 vs. T2)	0.60	1.86	50.69	0.32	0.747	[−3.14, 4.35]
Group × Time (control × T0)	−5.13	2.50	27.76	−2.05	0.050	[−10.25, −0.01]
Group × Time (control × T1)	−1.24	2.30	49.61	−0.54	0.594	[−5.86, 3.39]
Gender (female)	1.95	2.94	50.28	0.67	0.509	[−3.95, 7.85]
Ward (internal medicine)	0.05	2.69	49.74	0.02	0.984	[−5.34, 5.45]
Age	0.15	0.08	45.00	1.76	0.085	[−0.02, 0.31]
**Panel B Type III Tests of Fixed Effects**						
**Parameters**	**Num df**	**Den df**	**F**	***p*-value**		
Group	1	55.48	3.43	0.069		
Time	2	39.19	0.98	0.384		
Group × Time	2	39.82	3.13	0.055		
Gender	1	50.28	0.44	0.509		
Ward	1	49.74	0.00	0.984		
Age	1	45.00	3.11	0.085		
**C. Variance Components and AR (1) Parameter**						
Component	Estimate	Std. Error	95% CI			
Random-intercept variance	29.31	17.46	[9.12, 94.21]			
Residual variance	21.62	14.83	[5.63, 82.95]			
AR(1)	0.38	0.43	[−0.53, 0.88]			

Note CI = confidence interval; df = degrees of freedom; β = unstandardized coefficient; * *p* < 0.05. Reference categories: Group intervention; Time = T2 6 months; Gender = male; Ward = gynecology.

**Table 5:** Mixed-effects model results for the IPOS subscale emotional symptoms.

**A. Fixed-Effect Estimates**						
**Parameter**	**β**	**Std. Error**	**df**	**t-Value**	**Sig.**	**95% CI**
Intercept	5.90	1.76	71.98	3.35	0.001	[2.39, 9.41]
Group (control)	2.76	1.54	99.25	1.80	0.075	[−0.29, 5.81]
Time (T0 vs. T2)	0.30	1.36	40.59	0.22	0.825	[−2.45, 3.05]
Time (T1 vs. T2)	0.06	1.30	64.24	0.05	0.963	[−2.53, 2.65]
Group × Time (control × T0)	0.84	1.70	36.50	0.49	0.625	[−2.60, 4.28]
Group × Time (control × T1)	−0.70	1.62	60.09	−0.43	0.668	[−3.93, 2.54]
Gender (female)	−0.51	1.30	49.04	−0.39	0.699	[−3.11, 2.10]
Ward (internal medicine)	−0.58	1.18	50.54	−0.49	0.625	[−3.00, 1.80]
Age	0.02	0.04	38.81	0.45	0.653	[−0.06, 0.09]
**B. Type III Tests of Fixed Effects**						
**Parameters**	**Num df**	**Den df**	**F**	***p*-value**		
Group	1	49.20	10.44	0.002 *		
Time	2	54.31	1.33	0.272		
Group × Time	2	55.93	0.78	0.466		
Gender	1	49.04	0.15	0.699		
Ward	1	50.54	0.24	0.625		
Age	1	38.81	0.21	0.653		
**C. Variance Components and AR (1) Parameter**						
**Component**	**Estimate**	**Std. Error**	**95% CI**			
Random-intercept variance	0.37	0.43	[−0.53, 0.88]			
Residual variance	0.37	8.00	[0, 5.73 × 10^15^] **			
AR(1)	11.29	8.00	[2.83, 45.05]			

Note CI = confidence interval; df = degrees of freedom; β = unstandardized coefficient; * *p* < 0.05. Reference categories: Group = intervention; Time = T2 6 months; Gender = male; Ward = gynecology. ** Variance CIs are Wald-type (estimate ± 1.96 × SE), truncated at zero, with large upper bounds shown in scientific notation.

**Table 6:** Mixed-effects model results for ECOG performance score.

**Pannel A. Fixed-Effect Estimates**						
**Parameter**	**β**	**Std. Error**	**df**	**t-Value**	***p*-Value**	**95% CI**
Intercept	1.19	0.50	69.92	2.39	0.020 *	[0.20, 2.18]
Group (control)	0.78	0.37	85.60	2.09	0.040 *	[0.04, 1.51]
Time (T0 vs. T2)	0.45	0.28	36.51	1.61	0.115	[−0.12, 1.02]
Time (T1 vs. T2)	0.24	0.27	51.66	0.87	0.386	[−0.31, 0.79]
Group × Time (control × T0)	−0.55	0.34	34.35	−1.62	0.115	[−1.25, 0.14]
Group × Time (control × T1)	–0.43	0.33	50.51	–1.30	0.200	[−1.10, 0.24]
Gender (female)	−0.02	0.39	51.18	−0.04	0.970	[−0.80, 0.77]
Ward (internal medicine)	1.05	0.35	51.01	3.00	0.004 *	[0.35, 1.74]
Age	0.00	0.01	45.79	0.37	0.712	[−0.02, 0.03]
**Panel B. Type III Tests of Fixed Effects**						
**Parameters**	**Num df**	**Den df**	**F**	***p*-value**		
Group	1	56.03	3.04	0.087		
Time	2	44.00	0.95	0.394		
Group × Time	2	44.48	1.32	0.278		
Gender	1	51.18	0.00	0.970		
Ward	1	51.01	9.02	0.004 *		
Age	1	54.79	0.14	0.712		
**C. Variance Components and AR (1) Parameter**						
**Component**	**Estimate**	**Std. Error**	**95% CI**			
Random-intercept variance	0.54	0.19	[0.27, 1.09]			
Residual variance	0.32	0.13	[0.15, 0.71]			
AR(1)	0.19	0.34	[−0.45, 0.71]			

Note CI = confidence interval; df = degrees of freedom; β = unstandardized coefficient; * *p* < 0.05. Reference categories: Group = intervention; Time = T2 6 months; Gender = male; Ward = gynecology.

## Data Availability

The datasets used and/or analyzed during the current study are available from the corresponding author on reasonable request.

## Supplementary Materials

The following supporting information can be downloaded at: https://www.mdpi.com/article/10.3390/healthcare13162024/s1. STROBE Statement—checklist of items that should be included in reports of observational studies.

## Abbreviations

The following abbreviations are used in this manuscript:

AaR	Attrition at random
ADD	Attrition due to death
ADI	Attrition due to illness
CG	Control group
ECOG	Eastern Cooperative Oncology Group Performance Status
HCPs	Healthcare professionals
IG	Intervention group
ICC	Intraclass Correlation Coefficient
IPOS	Integrated Palliative Care Outcome Scale
LYOL-C-II	Last Year of Life Study Cologne—Part 2
M	Mean
MCS-12	Mental Component Score
MINI	Minimally Invasive Intervention
PCS-12	Physical Component Score
PROMS	Patient-reported outcome measures
QoL	Quality of life
QPS	Question prompt sheets
SF12	Short-Form Health Service
SD	Standard deviation
SPICT-DETM	Supportive and Palliative Care Indicators Tool
SQ	Surprise Question
T (0-4)	Timepoints

## Appendix A

**Table A1:** Average values for the physical and mental quality of life of control and intervention group, measured using the Short-Form Health Service Questionnaire (SF12).

	PCS		MCS	
	Control M (SD)	Intervention M (SD)	Control M (SD)	Intervention M (SD)
T0	31.34 (5.53)	33.40 (5.50)	38.63 (7.53)	38.09 (7.25)
T1	32.54 (6.51)	35.14 (7.20)	38.10 (7.06)	42.03 (4.82)
T2	29.24 (5.55)	29.74 (4.93)	39.68 (7.27)	44.67 (6.24)

**Table A2:** Mixed-effects model results of fixed parameters for the IPOS total score.

**A. Fixed-Effect Estimates**						
**Parameter**	**β**	**Std. Error**	**df**	**t-Value**	***p*-Value**	**95% CI**
Intercept	20.30	5.01	71.41	4.05	<0.001	[10.31, 30.29]
Group (control)	8.77	3.99	95.46	2.20	0.030 *	[0.854, 16.69]
Time (T0 vs. T2)	4.53	3.23	42.63	1.40	0.168	[−1.98, 11.04]
Time (T1 vs. T2)	2.22	2.89	56.43	0.77	0.445	[−3.56, 8.00]
Group × Time (control × T0)	−2.97	4.05	39.41	−0.74	0.476	[−11.15, 5.21]
Group × Time (control × T1)	−3.39	3.56	54.27	−0.95	0.346	[−10.53, 3.75]
Gender (female)	1.59	3.92	51.78	0.41	0.687	[−6.29, 9.46]
Ward (internal medicine)	−0.31	3.59	51.63	−0.09	0.932	[−7.51, 6.89]
Age	0.21	0.11	44.75	1.87	0.068	[−0.016, 0.43]
**B. Type III Tests of Fixed Effects**						
**Parameters**	**Num df**	**Den df**	**F**	***p*-value**		
Group	1	56.79	6.44	0.014 *		
Time	2	46.74	1.91	0.159		
Group × Time	2	47.68	0.45	0.638		
Gender	1	51.78	0.16	0.687		
Ward	1	51.63	0.01	0.932		
Age	1	44.75	3.50	0.068		
**C. Variance Components and AR (1) Parameter**						
**Component**	**Estimate**	**Std. Error**	**95% CI**			
Random-intercept variance	8.87	90.02	[0, 3.87× 10^9^] **			
Residual variance	85.90	89.54	[11.14, 666.55]			
AR(1)	0.62	0.39	[−0.48, 0.96]			

Note CI = confidence interval; df = degrees of freedom; β = unstandardized coefficient; * *p* < 0.05. Reference categories: Group = intervention; Time = T2 6 months; Gender = male; Ward = gynecology. ** Variance CIs are Wald-type (estimate ± 1.96 × SE), truncated at zero, with large upper bounds shown in scientific notation.

**Table A3:** Mixed-effects model results for the IPOS subscale communication and practical issues.

**A. Fixed-Effect Estimates**						
**Parameter**	**β**	**Std. Error**	**df**	**t-Value**	***p*-Value**	**95% CI**
Intercept	4.05	1.14	77.12	3.55	0.001	[1.78, 6.31]
Group (control)	1.09	1.02	95.46	1.07	0.288	[−0.94, 3.12]
Time (T0 vs. T2)	0.22	0.91	40.63	0.24	0.812	[−1.62, 2.06]
Time (T1 vs. T2)	1.29	0.94	60.59	1.36	0.177	[−0.59, 3.17]
Group × Time (control × T0)	0.51	1.13	37.23	0.45	0.657	[−1.78, 2.80]
Group × Time (control × T1)	−1.46	1.18	55.73	−1.24	0.222	[−3.83, 0.91]
Gender (female)	0.07	0.81	46.96	0.08	0.936	[−1.57, 1.70]
Ward (internal medicine)	−0.33	0.74	48.15	−0.45	0.656	[−1.82, 1.16]
Age	0.03	0.02	35.29	1.54	0.133	[−0.01, 0.08]
**B. Type III Tests of Fixed Effects**						
**Parameters**	**Num df**	**Den df**	**F**	***p*-value**		
Group	1	42.60	2.08	0.157		
Time	2	56.31	0.47	0.626		
Group × Time	2	57.67	2.44	0.096		
Gender	1	46.96	0.01	0.936		
Ward	1	48.15	0.20	0.656		
Age	1	35.29	2.36	0.133		
**C. Variance Components and AR (1) Parameter**						
**Component**	**Estimate**	**Std. Error**	**95% CI**			
Random-intercept variance	0.75	1.61	[0.01, 50.49]			
Residual variance	4.20	1.61	[1.98, 8.92]			
AR(1)	0.06	0.37	[–0.58, 0.65]			

Note CI = confidence interval; df = degrees of freedom; β = unstandardized coefficient; Reference categories: Group = intervention; Time = T2 6 months; Gender = male; Ward = gynecology.
